# The evolution of inflammatory mediators

**DOI:** 10.1155/S0962935196000014

**Published:** 1996-02

**Authors:** Andrew F. Rowley

**Affiliations:** 1 School of Biological Sciences University of Wales Swansea Singleton Park Swansea SA2 8PP UK

## Abstract

Invertebrates do not display the level of sophistication in immune reactivity characteristic of mammals and other ‘higher’ vertebrates. Their great number and diversity of forms, however, reflect their evolutionary success and hence they must have effective mechanisms of defence to deal with parasites and pathogens and altered self tissues. Inflammation appears to be an important first line defence in all invertebrates and vertebrates. This brief review deals with the inflammatory responses of invertebrates and fish concentrating on the cell types involved and the mediators of inflammation, in particular, eicosanoids, cytokines and adhesion molecules.


Mediators of Inflammation, 5, 3–13 (1996)

### The evolution of inflammatory mediators

**Andrew F. Rowley**

School of Biological Sciences, University of Wales, Swansea, Singleton Park, Swansea, SA2 8PP, UK. Fax: (+44) (1) 792 295447.

**INVERTEBRATES do not display the level of sophistication in immune reactivity characteristic of mammals and other 'higher' vertebrates. Their great number and diversity of forms, however, reflect their evolutionary success and hence they must have effective mechanisms of defence to deal with parasites and pathogens and altered self tissues. Inflammation appears to be an important first line defence in all invertebrates and vertebrates. This brief review deals with the inflammatory responses of invertebrates and fish concentrating on the cell types involved and the mediators of inflammation, in particular, eicosanoids, cytokines and adhesion molecules.**

**Key words:** Cytokines, Eicosanoids, Fish, Inflammation, Integrins, Invertebrates, Phenoloxidase system

### Introduction

It is ironic that one of the fathers of modern immunology, Elie Metchnikoff, carried out his most significant experiments in a non-mammalian model, the starfish larva. While working in the Mediterranean at the Straits of Messina he implanted rose thorns into transparent starfish larvae in which he was able to observe the behaviour of blood cells that surrounded the 'foreign' implant. From this simple observation he progressed to demonstrate the phagocytic activity of these cells towards bacteria and subsequently he applied this observation made in an invertebrate to explain the role of phagocytic leucocytes in humans during inflammation. Later in the 1920s the French scientist Metalnikov, a pupil of Metchnikoff, marvelled at the phenomenal ability of insect larvae to deal with injection of pathogenic bacteria that would overwhelm the natural defences of the mammalian immune system.[1]

From these early reports it can be seen that invertebrates and other 'lower' animals are capable of mounting highly effective inflammatory responses that at least match the capacity of any mammal. Indeed, invertebrates with their lack of immune system comprising lymphocytes and immunoglobulin, may therefore be more dependent on nonspecific defences such as inflammation to maintain their integrity. This brief review aims to introduce the reader to the nature of the inflammatory response in invertebrates and 'lower' vertebrates concentrating on the nature of the cells involved and the chemical mediators of this cellular response.

### The nature of the invertebrate inflammatory response and the cell types involved

Phagocytosis is a universal phenomenon within the animal kingdom that first functioned as a feeding mechanism in unicellular organisms such as amoebae and was later employed as a defensive mechanism to maintain the integrity of more complex multicellular organisms.[2] Phagocytic blood cells have been described in organisms from anthrozoans[3] through to the cephalochordates[4] and urochordates[5] that are believed to be the closest living relatives of the first vertebrates.[6] As shown in Fig. 1 early multicellular animals, whose modern day representatives include sponges and coelenterates, gave rise to more advanced forms with a body space referred to as a coelom. In most invertebrates the coelom is the main body cavity that is filled with fluid and cells termed coelomocytes. Not all animals have an extensive coelom and in insects, molluscs and urochordates for example, the main body cavity is a haemocoel and hence the cells are described as haemocytes rather than coelomocytes. Some invertebrates, including annelid worms (see Fig. 1) have both haemocytes in their blood vascular system and coelomocytes in the coelom.[2] Because all the main organs are bathed in either coelomic fluid or blood, cells are rapidly delivered to sites of damage or microbial invasion making the inflammatory response highly efficient.

*Classification and characterization of invertebrate 'blood' cells involved in inflammation*: For a detailed appraisal of the structure and classification of invertebrate leucocytes the reader is

© 1996 Rapid Science Publishers

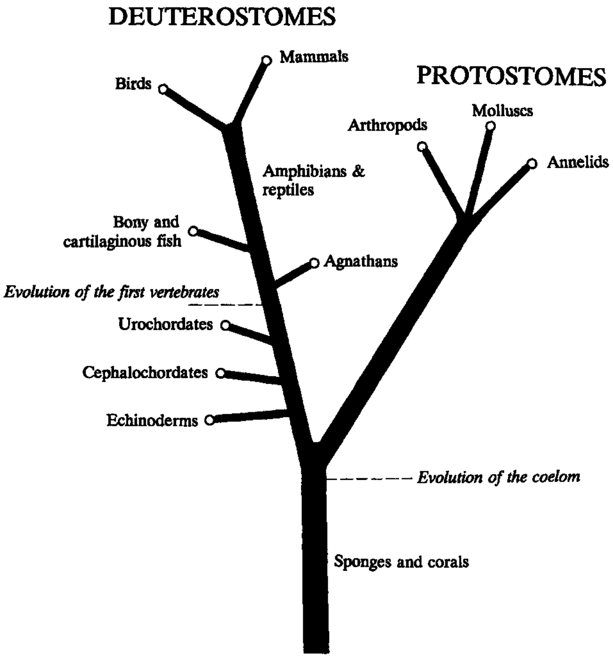

FIG. 1. Simplified evolutionary tree of the animal kingdom showing the main groups of animals referred to in the text.

directed to Ratcliffe and Rowley[7] or Ratcliffe *et al.*[8] As already mentioned, the cell type of principal interest in the context of inflammation is the phagocyte. This cell type has a multitude of names in the literature on invertebrate blood cells including amoebocyte, immunocyte, macrophage, hyaline cell, granulocyte, plasmatocyte and granular cell. It is important to stress that the use of nomenclature borrowed from mammalian haematology, such as granulocyte and macrophage, does not imply an evolutionary interrelationship. Indeed, it is unclear if the phagocytic blood cells of invertebrates gave rise to either the granulocytes, macrophages or both cell types in the vertebrates but it seems likely that the macrophage is the more ancient of these two groups of cells.[9] While most invertebrates have only a single class of phagocytic leucocytes, there are examples where morphologically distinct phagocytic cells exist in some animals. For example, in the bivalve mollusc, *Mytilus edulis*, both basophilic and eosinophilic granulocytes are phagocytic (Fig. 2).[10] Similarly, in *Ciona intestinalis* (a urochordate, see Fig. 1) hyaline and granular amoebocytes are both actively phagocytic towards foreign material.[5] Whether in these animals the two classes of phagocytic cells are part of a single maturation series, as in the case of mammalian monocytes and macrophages, or distinct cell types of different lineages, as with mammalian granulocytes and macrophages, is unknown. One approach that may resolve this question is to raise monoclonal antibodies to purified populations of leucocytes and use these as probes for ontogenetic studies. Such an approach has been made possible by the development of density gradient centrifugation techniques adapted for a range of invertebrate leucocytes (Fig. 2). To date monoclonalantibodies have been produced against crustacean,[11] molluscan,[12] insectan[13,14] and urochordate[15] leucocytes but they have not been employed to any great extent to study this problem of leucocyte interrelationships.

As well as the phagocytic leucocyte, mention should also be made of an additional cell type found mainly in arthropods (insects and crustaceans) that houses a range of mediators of inflammation in a similar way to the mast cell in mammals. Such cells are highly unstable and following wounding or contact with microbial material (e.g. LPS) they degranulate, releasing factors that influence the behaviour of phago-

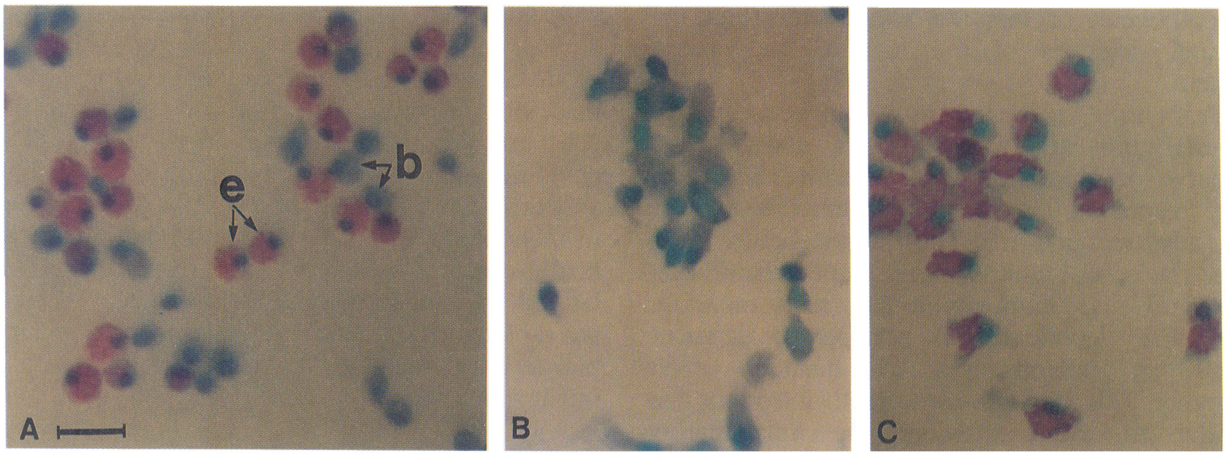

FIG. 2. *Mytilus edulis* basophilic (b) and eosinophilic (e) granulocytes before (A) and after (B and C) separation using density gradient centrifugation. Both the basophilic and eosinophilic granulocytes have been found to be phagocytic.[10] Scale bar = 20 μm. Micrograph courtesy of Dr E.A. Dyrynda.

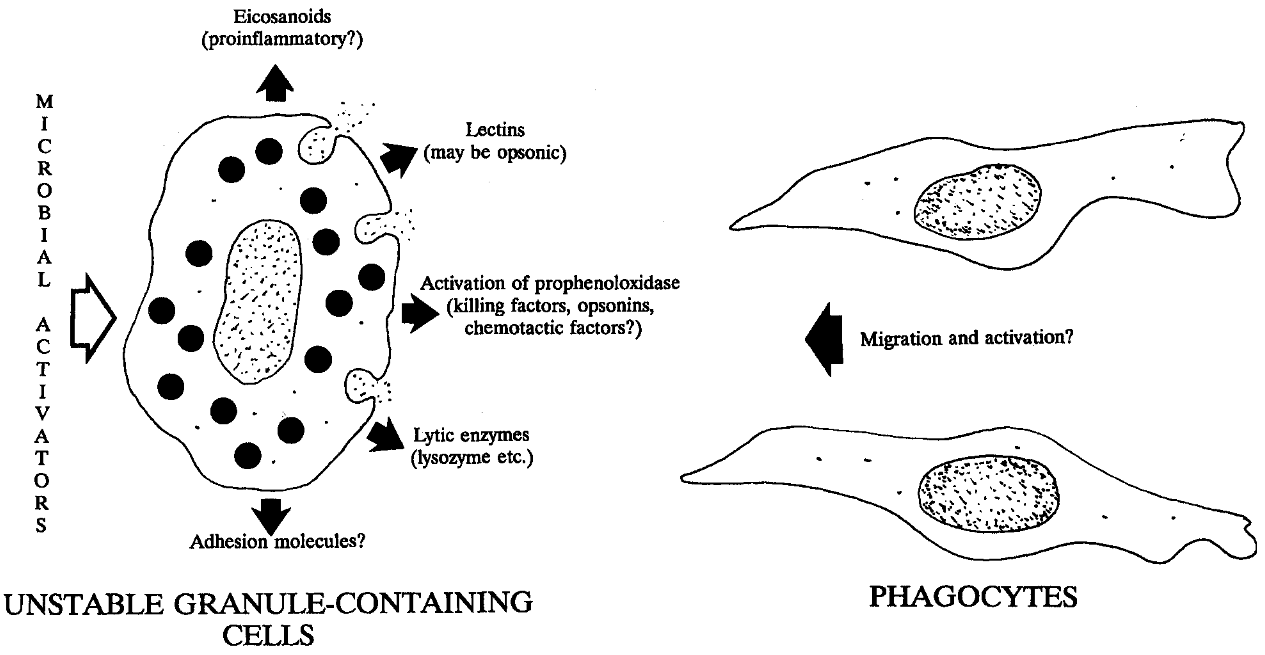

FIG. 3. Diagrammatic representation of the behaviour of unstable granule-containing cells and phagocytes during inflammatory responses in arthropods. For further details see the main text.

cytes (Fig. 3). This 'mast cell analogue' of invertebrates is referred to as a granular cell in crustaceans and cystocyte, coagulocyte or granular cell in insects.[8] The nature of some of these mediators is described later in this review.

*The inflammatory processes of invertebrates:* There are two main processes characteristic of the inflammatory response of invertebrates. The first is phagocytosis and essentially this defence reaction is very similar to its mammalian counterpart with the exception of the recognition process.[8] The mechanisms involved in the intracellular killing of phagocytosed material within invertebrate phagocytes involves various oxygen radicals formed during the respiratory burst,[16,17] NO generation[18,19] and hydrolytic enzymes including lysozyme.[20] The second defence reaction is one of encapsulation which bears some superficial similarity to granuloma formation in mammals. The encapsulation response is found during integumental wound healing, parasite invasion and bacterial challenge. In this latter case it is normally referred to as nodule formation or nodulation. Both nodule formation and encapsulation are biphasic processes in arthropods where these events have been researched extensively.[8] The first stage involves the degranulation of unstable cells to release various pro-inflammatory factors (Fig. 3). The final stage involves the ensheathment of this mass of cells by phagocytes, effectively walling-off invading parasites and microbial agents (Fig. 4A). During wound healing the first stage plugs the wound, hence limiting blood loss, and the proceeding ensheathment strengthens this haemostatic response and provides a framework for the regeneration of integumental tissues (Fig. 4B).

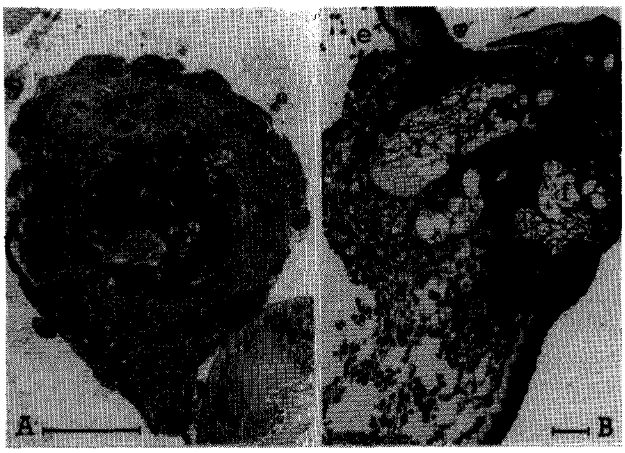

FIG. 4. (A) Section through a nodule formed in the haemocoel of an insect in response to the injection of bacteria. The central melanized mass consists of necrotic unstable granule-containing cells and entrapped bacteria. The outermost sheath (s) of flattened phagocytic cells walls off the bacteria hence stopping them gaining access to the rest of the insect. Scale bar = 50 μm. Micrograph courtesy of Professor N.A. Ratcliffe. (B). Cuticular wound through an insect showing the encapsulation response over this area. Note the sheath (s) of surrounding phagocytic cells that seals off the melanized 'scab' that consists of unstable granule-containing cells and extruded fat body (f). Ultimately, the epithelial cells (e), that secrete the cuticle, reform using the phagocyte sheath as a base to migrate over. Scale bar = 50 μm.

### Mediators of inflammation in invertebrates

*Cytokine-like molecules:* There is much evidence for the existence of pro-inflammatory cytokine-like molecules in both protostome and deuterostome invertebrates (Fig. 1). Prendergast and Liu[21] were the first to show that the starfish, *Asterias forbesi*, contains a cytokine-like factor in the 'blood' which stimulates monocyte chemotaxis and macrophage activation in mammals. Additionally, this 38 kDa molecule, appropriately named sea star factor (SSF), initiates an inflammatory-like response in *A. forbesi* where coelomocytes aggregate and ensheath pellets of polymer incorporating SSF implanted in the coelom.[22] A further 29.5 kDa IL-1-like molecule has also been isolated from starfish by Beck and Habicht[23] whose biological activity can be inhibited by polyclonal antisera to mammalian IL-1 implying some evolutionary sequence conservation of this molecule. IL-1α and IL-1β-like molecules have also been found in the urochordate, *Styela clava*.[24] This group of animals is of particular interest as they are closely related to the ancestors of the first vertebrates and hence may provide clues to the immunological complexity of pre-vertebrates. One fraction of >10 kDa obtained from the haemolymph of *S. clava* by gel filtration and chromatofocusing chromatography, stimulated the mitogenic proliferation of both *Styela* haemocytes and murine thymocytes, while the fraction containing IL-1α-like activity only had such activity with the murine cell type.[24,25] Further studies have shown that a 17.5 kDa protein from *Styela* with IL-1 activity also acts as an opsonin and chemoattractant in this animal.[26,27]

Much attention has focused on the presence and activity of cytokines in molluscs that as a group belong to the protostome lineage (see Fig. 1). Not only have a variety of cytokines been located immunocytochemically in the haemocytes of some molluscs[28] but lipopolysaccharide stimulation of the haemocytes from the bivalve mollusc, *Mytilus edulis*, causes the release of TNF and/or IL-1-like factors from these cells.[29] *Mytilus* blood cells also respond to rIL-1α and rTNF-α by changes in their adhesive behaviour[30] and IL-1 also stimulates the chemotactic activity of these cells.[31] The specificity of the reaction of *Mytilus* haemocytes to mammalian cytokines is also suggested by the inhibitory activity of polyclonal antibodies to either rIL-1α or TNF-α.

Mention should also be made of experiments that appear to demonstrate a link between the invertebrate neuroendocrine and immune systems that involves cytokine-like factors. Melanocyte-stimulating hormone, met-enkephalin and substance P antagonize the effect of TNF-α in *Mytilus* in which the expression of a neutral endopeptidase 24.11 appears to be involved.[32] Furthermore, generation of biogenic amines (epinephrine, dopamine and norepinephrine) by molluscan haemocytes is significantly reduced following stimulation with corticotrophin-releasing factor by preincubation of these cells with r-IL-α, IL-1β, TNF-α or TNF-β.[33] These, and other experiments, suggest that a link exists between the neuroendocrine and immune systems of invertebrates involving cytokines in a similar (homologous?) way to the situation in mammals.

In summary, there is much evidence to suggest that cytokines equivalent to their mammalian counterparts exist in invertebrates. A certain degree of caution should be expressed, however, in a too liberal interpretation of these results as we do not have available any sequence data to give insight into structural relationships between invertebrate and mammalian cytokines. The finding of apparent TNF-α immunoreactive material associated with molluscan haemocytes, using a polyclonal antibody to human TNF-α illustrates the problems with such an approach to identifying cytokines in invertebrates.[34] In the study they found that this polyclonal antibody reacted strongly with a 53 kDa molecule and weakly with a 120 kDa molecule associated with these cells that is unlike the 17 kDa mammalian TNF-α. Such findings serve to remind us that antibodies raised against human cytokines may react with unrelated molecules in animals widely separated by many millions of years of evolution. Alternatively, invertebrate cytokine-like activity may reside in molecules with very different structures to those in their mammalian counterparts. Only when we have purified and sequenced several invertebrate cytokine activities will answers to such questions become available.

*The prophenoloxidase-activating system:* The melanization response found in the haemocytic capsules and nodules formed in response to foreign agents is a common feature in arthropods (insects and crustaceans).[8,35] Over twenty years ago this association between melanin and inflammation was suggested to reflect a killing mechanism caused by the generation of toxic quinones intermediate in the formation of melanin.[36] Since this observation, the biochemistry of the cascade that yields melanin has been subject to detailed examination (see References 8, 35 and 37 for reviews). Central to this system is the enzyme phenoloxidase (EC 1.14.18.1) found inside haemocytes or in the plasma as a proenzyme (prophenoloxidase). This system is initially activated by microbial products, such as LPS or glucans, leading to cleavage of prophenoloxidase by serine protease activity. The end result of this pathway is the generation of a range of opsonic and haemostatic factors that influence the behaviour of other blood cells during inflammatory responses such as nodule formation, encapsulation and phagocytosis. Although the prophenoloxidase activating system has been likened to the vertebrate complement system,[37] the nature of some of the biologically active factors generated and their mode of generation is still unclear.

*Eicosanoids:* As eicosanoids, in particular leukotriene (LT) $B_4$, have been reported to play a central role in inflammation in mammals it is not surprising that attempts have been made to assess the potential of these compounds as mediators of inflammation in invertebrates. With the exception of arthropod venom,[38] invertebrates do not appear to express the 5-lipoxygenase and LTA hydrolase activities required for the generation of $LTB_4$.[39] Cyclooxygenases are, however, found in all invertebrates examined to date[39] and hence prostaglandins (PG) are potential candidates for playing a role in mediating inflammatory responses in these animals. Only a few studies have examined the eicosanoid generating capacity of invertebrate leucocytes. For example, crab (*Carcinus maenas*) blood cells can synthesize both lipoxygenase and cyclooxygenase derivatives including 8(*R*)-hydroxyeicosatetraenoic acid (8-HETE), 8(*R*)-hydroxyeicosapentaenoic acid (8-HEPE), $PGE_2$, and thromboxane $B_2$.[40] Similarly, in an insect, *Manduca sexta*, the haemocytes generate 15-HETE as their major product, with $PGE_2$, $PGD_2$, $PGF_{2\alpha}$ and $PGA_2$ as the main cyclooxygenase-derived products.[41] In both cases, inhibition of presumptive lipoxy-genase and cyclooxygenase-derived product generation can be achieved with specific inhibitors.[40,41]

Recently reported experiments have given insight into the possible role of eicosanoids as inflammatory mediators in insects. These studies used nodule formation as the assay system where insect larvae of the tobacco hornworm (*M. sexta*) were injected with bacteria (*Serratia marcescens*) and the size and number of nodules formed in the haemocoel in response to this particulate insult determined.[42] They found that prior injection of the phospholipase $A_2$ ($PLA_2$) inhibitor, dexamethasone, caused a dose-dependent inhibition of the nodule formation response to *S. marcescens*. As $PLA_2$ is required for the provision of free fatty acid precursors for eicosanoid biosynthesis, some insects were also injected with dexamethasone together with arachidonic or eicosapentaenoic acids (both substrates for eicosanoid generation). These fatty acid 'rescue' experiments reversed the effect of dexamethasone showing the specificity of the activity of this $PLA_2$ inhibitor. Similarly, a range of lipoxygenase and cyclooxygenase inhibitors also reduced the number of nodules formed in response to bacterial challenge.[42] Hence, there is clear evidence for a role of eicosanoids in this inflammatory response although it remains to be determined which products are involved and the mechanism of their action is also unknown. There are several stages of nodule formation during which eicosanoids could participate. The initial stage involves the degranulation of unstable cells to release pre-formed mediators (e.g. lectins) together with the biosynthesis of additional factors (e.g. prophenoloxidase-derived products and pro-inflammatory eicosanoids?) (Fig. 3). The second stage involves the ensheathment of this mass of degranulated cells by phagocytes to form a mature nodule. Potentially, eicosanoids could be involved in this second stage by modifying the adhesive properties of phagocytes or initiating their migrative behaviour to allow them to become incorporated in the nodules.

*Adhesion molecules:* Although some adhesion molecules have been characterized in invertebrates little was known until recently about their role in inflammation. The integrin family of cell surface adhesion molecules appears to have a long evolutionary history and examples of these molecules have been found in *Drosophila*[43] and the freshwater crayfish, *Pacifastacus leniusculus*.[44] In this latter example, the integrin-like molecule has been found to contain the characteristic RGD sequence (Arg–Gly–Asp) that represents the functional binding site to its ligand(s).[44,45] The *P. leniusculus* adhesion molecule is a 76 kDa protein that causes degranulation of crayfish granular blood cells[44,46] thereby causing the activation of the prophenoloxidase system (see Fig. 3) which in turn promotes encapsulation activity.[47] A similar factor has also been found in another crustacean, the crab, *Carcinus maenas*.[48] This 80 kDa protein found in the granular haemocytes acts as an opsonin enhancing the phagocytic ability of hyaline blood cells of this animal.[48] This protein, and its equivalent in other species, may be the opsonic principle generated by the prophenoloxidase system in insects and crustaceans during the degranulation response in unstable granule-containing haemocytes. Further evidence for the role of integrins in the defence reactions of invertebrates comes from recent interesting studies where Sepharose beads were conjugated to the peptide, RGDS, and then incubated with haemocytes from the moth, *Pseudoplusia includens*.[49] Beads conjugated to this peptide were encapsulated by these blood cells while beads without peptide or beads with RGES were not ensheathed. Furthermore, soluble RGDS, but not RGES, inhibited this encapsulation response. These results imply that encapsulation involves an adhesion molecule with the characteristic RGD recognition motif.

Insight into a further potential adhesion molecule and its function in inflammation comes from the use of a monoclonal antibody raised against the haemocytes of the wax moth, *Galleria mellonella*.[50] This antibody reacts with an approx. 100 kDa molecule found associated with the unstable granular haemocytes that play a role in the first stage of nodule formation.[8] Blocking the action of the protein with this monoclonal antibody causes a reduction in the adhesion of wax moth haemocytes to glass substrates and nodule formation *in vivo*.[49] Unfortunately, the structure of the 100 kDa protein remains to be determined and so no sequence comparisons with other adhesion molecules can be made.

### The nature of the inflammatory response in 'lower' vertebrates and the cell types involved

The most significant stage in the evolution of the immune system came about with the appearance of the first vertebrates. These were probably the first animals with 'true' lymphocytes, with the ability to respond by clonal selectivity upon challenge. Furthermore, these animals would have had the ability to synthesize 'true' immunoglobulins (i.e. molecules with variable regions) that specifically interact with non- or altered-self materials. The modern day ancestors of these first vertebrates are fish (Fig. 1). The earliest vertebrates were jawless (agnathous) fish and the only animals to retain this feature are lampreys and hagfishes, thought to be the ancestors of some of the early vertebrates. All other fish are jawed and these include the two main divisions of cartilaginous (e.g. sharks, rays) and bony forms (e.g. trout, carp etc.). Hence fish are a useful group to examine in this review on the phylogeny of inflammation and the following sections highlight the changes brought about in the inflammatory response with their evolution from invertebrate ancestors.

*Leucocyte types involved in inflammation:* Not only did the lymphocyte probably make its first appearance during the evolution of the vertebrates but the other leucocyte types characteristic of mammalian blood, i.e. granulocytes and monocytes/macrophages, also evolved at this stage. Hence all vertebrates have remarkably similar leucocyte types reflecting a close association and a common ancestry. The only possible exception may be mast cells, although some fish have cells in the stratum compactum of the alimentary canal (Fig. 5), the dermis, gills and swimbladder with similar structural and functional properties to mammalian mast cells.[51,52] Mast cells have been clearly identified in all other vertebrates.

An area of some controversy is that of granulocyte heterogeneity in fish. Some species of fish have been reported to have neutrophilic, eosinophilic and basophilic granulocytes in peripheral blood, yet others may only have a single morphological type. Even more curious is the observation that at different stages in the life cycle of the same species there may be different types of granulocytes present. An example of this is in the lamprey (*Lampetra fluviatilis*) where both neutrophilic and eosinophilic granulocytes are found in the larval stage yet in the adult only the former cell type is present in the blood stream (Fig. 6).[53] In cartilaginous fish there may also be several different morphological types of eosinophilic granulocytes in the same species (Fig. 6), some of which appear to function in a manner equivalent to mammalian neutrophils.[53–55] Two main conclusions can be drawn from these findings. Firstly, the staining characteristics of fish granulocytes (i.e. eosinophilic, basophilic etc.) do not

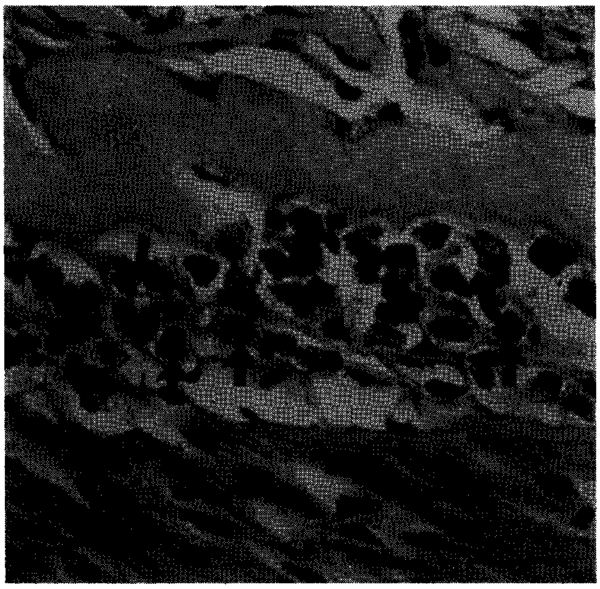

FIG. 5. Histological appearance of cells (unlabelled arrows) in the intestine of the rainbow trout, *Oncorhynchus mykiss*, thought to be functionally analogous to the mast cells of higher vertebrates. Scale bar = 50 μm.

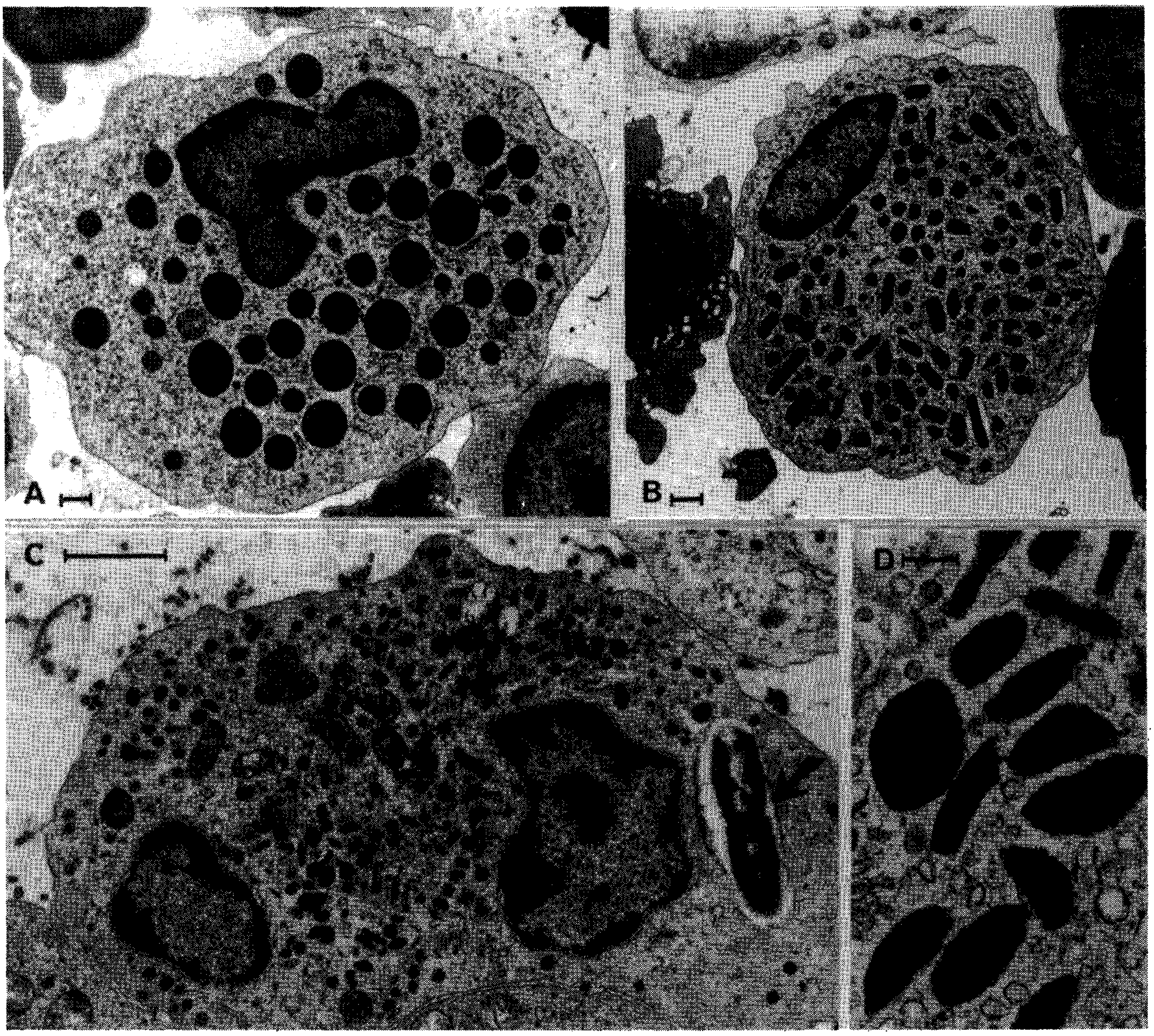

FIG. 6. Electron micrographs showing the structural diversity of fish granulocytes. (A, B). Eosinophilic granulocytes in the dogfish, *Scyliorhinus canicula*. (C). Neutrophilic granulocyte from the adult lamprey, *Lampetra fluviatilis*, containing a bacterium (unlabelled arrow) at an early stage of ingestion. (D). Granule sub-structure in neutrophil of *L. fluviatilis*. Scale bars = 1 μm (A–C) and 0.2 μm (D).

always mirror functional diversity and secondly, that there is no common evolutionary trend in granulocyte heterogeneity within the different types of fish.

*The inflammatory response in fish:* This has been the subject of recent excellent reviews[56,57] and hence this following section is only included to present some more recent findings and highlight topics of particular interest to the reader. There are many descriptions of inflammatory exudate formation in fish following experimental challenge or in naturally infected fish. Several similarities exist between the mammalian and piscine acute inflammatory responses. For example, the cellular involvement in inflammation in fish appears to be biphasic with an influx of granulocytes followed by a later arrival of monocytes/macrophages.[56] Both cell types are also actively phagocytic.[57] Some key differences do exist, however, between the response in fish and mammals, in particular the dynamics and intensity of this reaction. Most studies have reported a protracted inflammatory response in fish where peak numbers of granulocytes and macrophages occur at about 1–2 days and 2–7 days respectively post-challenge.[56,58] In carp, the granulocytes that migrate to sites of inflammation originate from the head-kidney,[56] a haemopoietic tissue equivalent to mammalian bone marrow,[55] while the macrophages in exudates appear to originate from blood-derived monocytes. The site of monocytopoiesis in bony fish is usually the head-kidney and/or spleen.[55] Once at the site of inflammation, macrophages may become stimulated with increased phagocytic potential and enhanced antimicrobial activity.[59]

As would be expected, the sequence of events during phagocytosis (chemotaxis, attachment, ingestion and intracellular digestion) are essentially the same in fish as in mammals.[57] The attachment of foreign material to both piscine granulocytes and mononuclear phagocytes appears to be aided by immunoglobulin[60–62] and complement fragments[63] at least in some cases, while subsequently the respiratory burst leads to an increase in oxygen radical generation.[57,64] There is also evidence for the production of NO in fish phagocytes that is enhanced by exposure to microbial products.[65,66] Various hydrolytic enzymes are present in lysosomes and granules in granulocytes and mononuclear phagocytes[67] and these presumably play a role in killing and digestion of ingested microorganisms.

### Inflammatory mediators in fish

*Cytokines:* Although IL-1-like cytokines have been demonstrated in fish nothing is known about their potential involvement in inflammatory responses. IL-1 has been shown to be generated by monocytes in the catfish, *Ictalurus punctatus*,[68] and by macrophages and granulocytes from the carp, *Cyprinus carpio*.[69] In catfish, polyclonal antisera to human IL-1α and IL-1β revealed immunopositive bands in Western blots of monocyte supernatants at 60, 43 and 30 kDa with IL-1α antiserum, and 70 and 21 kDa with IL-1β antiserum.[70] In carp, polyclonal antiserum to human rIL-1α reacted with a 22.3 kDa protein in Western blots of SDS–PAGE separated macrophage lysates while a 21.7 kDa band was revealed using antiserum against IL-1β.[69] A further immunoreactive 15 kDa band was found with both IL-1α and IL-1β antisera. Importantly, these antisera to IL-1α and IL-1β ablated the biological activity of supernatants from stimulated carp macrophages in the assays employed for IL-1 activity showing that at least one of the three proteins that interacted with the antibodies corresponds to the active principle in these preparations.[69]

Significant progress has been made in elucidating macrophage activating cytokines in fish largely as a result of Secombes and co-workers (see Reference 71 for review). Macrophage activating factor (MAF) can be generated by incubating head-kidney leucocytes with T cell mitogens and the cell type directly responsible in its generation belongs to a population of surface $Ig^-$ lymphocytes (T cell-like).[72] Activity resides in 19 and 32 kDa fractions although the active principle(s) have not been isolated and sequenced to date.[71] Several pieces of evidence suggest that MAF is a form of IFN-γ although the biological activity of fish MAF cannot be replaced by human IFN-γ.[71] The biological properties of MAF include the elevation in a number of macrophage activities including phagocytosis, adherence and spreading to substrates, respiratory burst and bacterial killing.[71] Recent studies have reported synergism between human rTNF-α and fish-derived MAF in terms of respiratory burst activity in fish macrophages.[73] This effect could be inhibited by prior incubation of target macrophages with monoclonal antibodies to the mammalian receptor for TNF-α.[73]

*Adhesion molecules:* The explosion in our understanding of the nature and function of adhesion molecules in the control of leucocyte migration during inflammation in mammals does not appear to have stimulated the search for similar molecules in fish and other lower vertebrates. Indeed, the only apparent report of a potential adhesion molecule in fish comes from a study on fish brain where 'foamy' macrophages were found to react with an antiserum to the $\beta_2$ chain of human leucocyte integrins.[74]

*Complement:* Although there is some evidence for the existence of complement components and associated factors in invertebrates, the evolution of the 'fully-functional' pathways of this system appears to coincide with the appearance of fishes. Hence, with the exception of possibly agnathous and cartilaginous fish, both classical and alternative pathways of complement activation exist in lower vertebrates.[75] There are several reports of both chemotactic/chemokinetic[76] and opsonic[77,78] activities for complement factors in fish, showing that these are important pro-inflammatory molecules in such animals.

*Eicosanoids:* The eicosanoid-generating ability of fish leucocytes has received a great deal of attention in the last decade. Most studies have concentrated on the biosynthetic capacity for eicosanoid generation in macrophages. For example, macrophages from the head-kidney of a number of species of fish have been found to contain 5- and 12-lipoxygenase activities that result in the synthesis of leukotrienes and lipoxins from endogenous or exogenous arachidonic (20:4,n-6) and eicosapentaenoic (20:5,n-3) acids.[79–82] Challenge of macrophages from the head-kidney of the rainbow trout, *Oncorhynchus mykiss*, with microbial factors (LPS, glucans) or calcium ionophore results in the rapid synthesis of both lipoxygenase and cyclooxygenase products including 12-HETE, 12-HEPE, lipoxin (LX) $A_4$, $LXA_5$, $LTB_4$, $LTB_5$ and $PGE_2$. Although this overall profile of products is similar to that found in mammalian macrophages it differs in the magnitude of the amounts generated. The lipoxin generating capacity of rainbow trout macrophages is about four to five times greater than reported in mammalian macrophages under the same conditions.[79] The greater amounts of lipoxin formed in some species of fish has led to the rather simplistic suggestion that these compounds may be more important in fish than in mammals.[79] Recently, an 18 kDa protein that appears to be the forerunner of 5-lipoxygenase activating protein (FLAP), has been found in lysates of trout macrophages.[83] FLAP has been found to be essential for cellular leukotriene biosynthesis in a range of mammalian leucocyte types.[84]

Several of the main eicosanoids generated by fish leucocytes have been found to be involved in inflammatory responses in these animals. Evidence supporting this viewpoint comes from both *in vivo* and *in vitro* experiments. Intraperitoneal injection of microbial products, such as zymosan or adjuvant, into fish results in an influx of leucocytes into the main body cavity with the typical protracted dynamics already described. Aspiration of the exudate formed followed by quantification of eicosanoid levels by enzyme immunoassay has demonstrated significant increases in the amounts of $LXA_4$, $LTB_4$ and $PGE_2$ (Fig. 7A).[85] In rainbow trout infected with proliferative kidney disease the causative agent, probably a myxosporean parasite, multiplies in the kidney, leading to a strong inflammatory response characterized by an increase in the number of macrophages.[86] Biopsies of infected kidney show a significant increase in $PGE_2$ activity in comparison with that in normal, uninfected kidney tissue (Fig. 7B). While the increase in the amounts of eicosanoids found *in vivo* suggests an involvement of these compounds in inflammation, it could also be argued that it simply reflects the increase in leucocyte numbers in such tissues mediated by other factors such as complement. However, the finding that injection of nordihydroguaiaretic acid, a lipoxygenase inhibitor, significantly reduces the number of macrophages and granulocytes in the peritoneal cavity of trout following challenge with the bacterium *Aeromonas salmonicida*, implies an active role of some eicosanoids in inflammation.[87]

As summarized in Table 1, eicosanoids modify and mediate a number of inflammatory processes of fish as assessed with *in vitro* assays. For example, $LTB_4$ has been shown to cause the migration of fish leucocytes *in vitro*. In the case of a cartilaginous fish, the lesser spotted dogfish, *Scyliorhinus canicula*, $LTB_4$ causes a dose-dependent increase in the migration of eosinophilic granulocytes in a migration under agarose assay.[88] Whether this reaction is chemotactic or

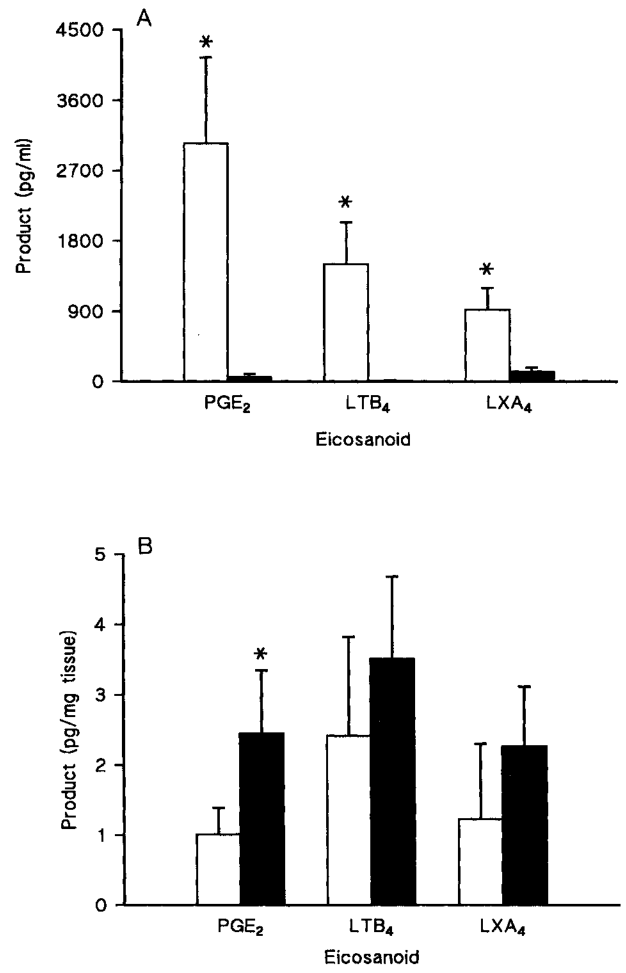

FIG. 7. Eicosanoid levels in experimentally induced and naturally infected trout tissues. (A). Levels of $PGE_2$, $LTB_4$ and $LXA_4$ in inflammatory exudates from trout injected 18 h previously with either zymosan □, or saline alone ■. (B). Eicosanoid levels in kidney biopsies from trout uninfected or infected with proliferative kidney disease. In both cases, the eicosanoid levels were quantified by enzyme immunoassay. Values shown are means ± S.D., $n = 3$, $^*p < 0.05$. Data from Reference 85. □, Uninfected; ■, infected.

chemokinetic in nature could not be ascertained with this method. Experiments have been carried out to examine the migration-inducing activities of $LTB_4$ and lipoxins for trout neutrophils using a Boyden chemotaxis chamber and checkerboard assays.[89] These allow for differentiation between chemokinesis and chemotaxis not possible in most other assays. $LXA_4$ was found to be one to three times more potent at inducing migration of trout neutrophils than $LTB_4$ at all concentrations tested ($0.03 - 1 \times 10^{-5}$ M). However, $LTB_4$ was found to be a chemotactic agent for trout neutrophils while $LXA_4$ was only a chemokinetic factor. In mammals, $LXA_4$ is regarded as an inhibitor of granulocyte migration in response to either FMLP or $LTB_4$.[90,91] Similarly, $LXA_4$ also inhibits human neutrophil transmigration through epithelial and endothelial cells.[92,93] These results suggest that in mammals, $LXA_4$

**Table 1.** Role of eicosanoids in *in vitro* inflammatory responses in fish

| Feature | Fish | $LXA_4$ | $LTB_4$ | 12-HETE | $PGE_2$ | References |
|---|---|---|---|---|---|---|
| Phagocytic uptake | Trout (*O. mykiss*) | — | — | Stimulation | Stimulation | 94 |
| Enzyme release during phagocytosis | Trout | Stimulation | — | — | Inhibition | 85 |
| Respiratory burst activity | Trout | NT | NT | NT | Inhibition | 95 |
| Leucocyte migration | Trout | Stimulation (chemokinetic) | Stimulation (chemotactic) | NT | NT | 89 |
| | Dogfish (*S. canicula*) | NT | Stimulation | NT | NT | 88 |

Key: —, No effect; NT, not tested.

inhibits several key events during inflammatory responses, while in fish it appears to be pro-inflammatory at least in the absence of other chemotactic agents. $PGE_2$ is the only other eicosanoid to be studied in detail in terms of its potential involvement in the nonspecific cellular defences of fish (Table 1). It is rather paradoxical that $PGE_2$ is a potent stimulator of the uptake of yeast particles by macrophages,[94] yet in the same cell type it inhibits both respiratory burst activity[95] and degranulation[85] that follow the ingestion process.

### References

1. Metalnikov S. Phagocytose et réactions des cellules dans l'immunité. *Ann Inst Pasteur Paris* 1924; **38**: 787–826.
2. Dales RP. Aspects of the evolution and development of body cavities, circulatory systems and "blood cells". In: Ratcliffe NA, Rowley AF, eds. *Invertebrate Blood Cells* Vol I. London: Academic Press, 1981; 3–16.
3. Patterson MJ, Landolt ML. Cellular reaction to injury in the anthrozoan *Anthopleura elegantissima. J Invertebr Pathol* 1979; **33**: 189–196.
4. Rhodes CP, Ratcliffe NA, Rowley AF. Presence of coelomocytes in the primitive chordate amphioxus (*Branchiostoma lanceolatum*). *Science NY* 1982; **217**: 263–265.
5. Rowley AF. The blood cells of the sea squirt, *Ciona intestinalis*: morphology, differential counts and *in vitro* phagocytic activity. *J Invertebr Pathol* 1981; **37**: 91–100.
6. Kardong KV. *Vertebrates, Comparative Anatomy, Function, Evolution.* Dubuque: Wm. C. Brown Publishers. 1995; 777.
7. Ratcliffe NA, Rowley AF. *Invertebrate Blood Cells* Vols 1 & 2. London: Academic Press, 1981; 641.
8. Ratcliffe NA, Rowley AF, Fitzgerald SE, Rhodes CP. Invertebrate immunity: basic concepts and recent advances. *Int Rev Cytol* 1985; **97**: 183–329.
9. Auger MJ, Ross JA. The biology of the macrophage. In: Lewis CE, McGee JO'D. *The Natural Immune System. The Macrophage.* Oxford: IRL Press, 1992; 1–74.
10. Pipe R, Coles JA, Farley S. Personal communication.
11. Rodriguez J, Boulo V, Mialhe E, Bachère E. Characterisation of shrimp hemocytes and plasma components by monoclonal antibodies. *J Cell Sci* 1995; **108**: 1043–1050.
12. Noël D, Pipe R, Elston R, Bachère E, Mialhe E. Antigenic characterization of hemocyte subpopulations in the mussel *Mytilus edulis* by means of monoclonal antibodies. *Mar Biol* 1994; **119**: 549–556.
13. Mullett H, Ratcliffe NA, Rowley AF. The generation and characterisation of anti-insect blood cell monoclonal antibodies. *J Cell Sci* 1993; **105**: 93–100.
14. Willott E, Trenczek T, Thrower LW, Kanost MP. Immunochemical identification of insect hemocyte populations: monoclonal antibodies distinguish four major hemocyte types in *Manduca sexta. Eur J Cell Biol* 1994; **65**: 417–423.
15. Uyama T, Nishikata T, Satoh N, Michibata H. Monoclonal antibodies specific to signet ring cells, and the vanadocytes of the tunicate, *Ascidia sydniensis samea. J Exp Zool* 1991; **259**: 196–201.
16. Pipe R. Generation of reactive oxygen metabolites by the haemocytes of the mussel, *Mytilus edulis. Dev Comp Immunol* 1992; **16**: 111–122.
17. Adema CM, Van der Knaap WPW, Sminia T. Molluscan hemocyte-mediated cytotoxicity: the role of reactive oxygen intermediates. *Rev Aquat Sci* 1991; **4**: 201–223.
18. Radomski MW, Martin JF, Moncada S. Synthesis of nitric oxide by the hemocytes of the American horseshoe crab (*Limulus polyphemus*). *Phil Trans Roy Soc London* 1991; **334**: 129–133.
19. Conte A, Ottaviani E. Nitric oxide synthase activity in molluscan hemocytes. *FEBS Lett* 1995; **365**: 120–124.
20. Cheng TC, Rodrick GE, Foley DA, Koehler SA. Release of lysozyme from hemolymph cells of *Mercenaria mercenaria* during phagocytosis. *J Invertebr Pathol* 1975; **25**: 261–265.
21. Prendergast RA, Liu SH. Isolation and characterization of sea star factor. *Scand J Immunol* 1976; **5**: 873–880.
22. Prendergast RA, Lutty GA, Scott AL. Directed inflammation: the phylogeny of lymphokines. *Dev Comp Immunol* 1983; **7**, 629–632.
23. Beck G, Habicht GS. Isolation and characterization of a primitive IL-1-like protein from an invertebrate. *Proc Natl Acad Sci USA* 1986; **83**: 7429–7433.
24. Raftos DA, Cooper EL, Habicht GS, Beck G. Invertebrate cytokines: tunicate cell proliferation stimulated by an interleukin 1-like molecule. *Proc Natl Acad Sci USA* 1991; **88**: 9518–9522.
25. Beck G, Vasta GR, Marchalonis JJ, Habicht GS. Characterization of interleukin-1 activity in tunicates. *Comp Biochem Physiol* 1989; **92B**: 93–98.
26. Kelly KL, Cooper EL, Raftos DA. Cytokine-like activities of a humoral opsonin from the solitary urochordate *Styela clava. Zool Sci* 1993; **10**: 57–64.
27. Cooper EL, Raftos DA, Zhang Z, Kelly KL. Purification and characterization of tunicate opsonins and cytokine-like proteins. In: Stolen JS, Fletcher TC, Smith SA, Zelikoff JT, Anderson RS, Söderhäll K, Weeks-Perkins BA, eds. *Techniques in Fish Immunology-4, 1995. Immunology and Pathology of Aquatic Invertebrates.* Fair Haven: SOS Publications, 1995; 43–48.
28. Ottaviani E, Franchini A, Franceschi C. Presence of several cytokine-like molecules in molluscan hemocytes. *Biochem Biophys Res Comm* 1993; **195**: 984–988.
29. Hughes TK, Smith EM, Barnett JA, Charles R, Stefano GB. LPS stimulated invertebrate hemocytes: a role for immunoreactive TNF and IL-1. *Dev Comp Immunol* 1991; **15**: 117–122.
30. Hughes TK, Smith EM, Chin R, *et al.* Interaction of immunoactive monokines (interleukin 1 and tumor necrosis factor) in the bivalve mollusc *Mytilus edulis. Proc Natl Acad Sci USA* 1990; **87**: 4426–4429.
31. Stefano GB, Smith EM, Cadet P, Hughes Jr TK. HIV gp120 alteration of DAMA and IL-1α induced chemotaxic responses in human and invertebrate immunocytes. *J Neuroimmunol* 1993; **43**: 177–184.
32. Stefano GB, Paemen LR, Hughes Jr TK. Autoimmunoregulation: differential modulation of CD10/neutral endopeptidase 24.11 by tumor necrosis factor and neuropeptides. *J Neuroimmunol* 1992; **41**: 9–14.
33. Ottaviani E, Caselgrandi E, Franceschi C. Cytokines and evolution: effects of IL-1α, IL-1β, TNF-α and TNF-β on the ancestral type of stress response. *Biochem Biophys Res Comm* 1995; **207**: 288–292.
34. Ouwe-Missi-Oukem-Boyer O, Porchet E, Capron A, Dissous C. Characterization of immunoreactive TNFα molecules in the gastropod *Biomphalaria glabrata. Dev Comp Immunol* 1994; **18**: 211–218.
35. Söderhäll K, Cerenius L, Johansson MW. The prophenoloxidase activating system and its role in invertebrate defense. *Ann NY Acad Sci* 1994; **712**: 155–161.
36. Taylor RL. A suggested role for the polyphenol–phenoloxidase system in invertebrate immunity. *J Invertebr Pathol* 1969; **14**: 427–428.
37. Söderhäll K. Prophenoxidase activating system and melanization—a recognition system of arthropods? A review. *Dev Comp Immunol* 1982; **6**: 601–611.
38. Czarnetzki BM, Thiele T, Rosenbach T. Evidence for leukotrienes in animal venoms. *J Allergy Clin Immunol* 1990; **85**: 505–509.
39. Rowley AF. Eicosanoids: aspects of their structure, function and evolution. In: Warr G, Cohen N, eds *Phylogenesis of Immune functions*. Boca Raton: CRC Press Inc., 1991; 269–294.
40. Hampson AJ, Rowley AF, Barrow SE, Steadman R. Biosynthesis of eicosanoids by blood cells of the crab, *Carcinus maenas*. *Biochim Biophys Acta* 1992; **1124**: 143–150.
41. Gadelhak GG, Pedibhotla VK, Stanley-Samuelson DW. Eicosanoid biosynthesis by hemocytes from the tobacco hornworm, *Manduca sexta*. *Insect Biochem Molec Biol* 1995; **25**: 743–749.
42. Miller JS, Nguyen T, Stanley-Samuelson DW. Eicosanoids mediate insect nodulation responses to bacterial infections. *Proc Natl Acad Sci USA* 1994; **91**: 12418–12422.
43. Gotwals PJ, Painesaunders SE, Stark KA, Hynes RO. *Drosophila* integrins and their ligands. *Curr Opinion Cell Biol* 1994; **6**: 734–739.
44. Johansson MW, Söderhäll K. A peptide containing the cell adhesion sequence RGD can mediate degranulation and cell adhesion of crayfish granular haemocytes *in vitro*. *Insect Biochem* 1989; **19**: 573–579.
45. Hynes RO. Integrins: versatility, modulation, and signaling in cell adhesion. *Cell* 1992; **69**: 11–25.
46. Johansson MW, Söderhäll K. A cell adhesion factor from crayfish haemocytes has degranulating activity towards crayfish granular cells. *Insect Biochem* 1989; **19**: 183–190.
47. Kobayashi M, Johansson MW, Söderhäll K. The 76kD cell-adhesion factor from crayfish haemocytes promotes encapsulation *in vitro*. *Cell Tissue Res* 1990; **260**: 113–118.
48. Thörnquist P-O, Johansson MW, Söderhäll K. Opsonic activity of cell adhesion proteins and β-1,3-glucan binding proteins from two crustaceans. *Dev Comp Immunol* 1994; **18**: 3–12.
49. Pech LL, Strand MR. Encapsulation of foreign targets by hemocytes of the moth *Pseudoplusia includens* (Lepidoptera: Noctuidae) involves an RGD-dependent cell adhesion mechanism. *J Insect Physiol* 1995; **41**: 481–488.
50. Mullett H, Ratcliffe NA, Rowley AF. Analysis of immune defences of the wax moth, *Galleria mellonella*, with anti-haemocytic monoclonal antibodies. *J Insect Physiol* 1993; **39**: 897–902.
51. Vallejo Jr AN, Ellis AE. Ultrastructural study of the response of eosinophil granule cells to *Aeromonas salmonicida* extracellular products and histamine liberators in rainbow trout *Salmo gairdneri* Richardson. *Dev Comp Immunol* 1989; **13**: 133–148.
52. Reite OB, Evensen O. Mast cells in the swimbladder of Atlantic salmon *Salmo salar*—histochemistry and responses to compound 48/80 and formalin-inactivated *Aeromonas salmonicida*. *Dis Aquatic Organism* 1994; **20**: 95–100.
53. Rowley AF, Page M. Ultrastructural, cytochemical and functional studies on the eosinophilic granulocytes of larval lampreys. *Cell Tissue Res* 1985: **240**: 705–709.
54. Mainwaring G, Rowley AF. Studies on granulocyte heterogeneity in elasmobranchs. In: Manning MJ, Tatner MF, eds. *Fish Immunology*. London: Academic Press, 1985; 57–69.
55. Rowley AF, Hunt TC, Page M, Mainwaring G. Fish. In: Rowley AF, Ratcliffe NA, eds. *Vertebrate Blood Cells*. Cambridge: Cambridge University Press, 1988; 19–127.
56. Suzuki Y, Iida T. Fish granulocytes in the process of inflammation. *Annu Rev Fish Disease* 1992; **2**: 73–89.
57. Secombes CJ, Fletcher TC. The role of phagocytes in the protective mechanisms of fish. *Annu Rev Fish Disease* 1992; **2**: 53–71.
58. MacArthur JI, Fletcher TC, Pirie BJS, Davidson BJL, Thomson AW. Peritoneal inflammatory cells in plaice, *Pleuronectes platessa* L.: effects of stress and endotoxin. *J Fish Biol* 1984; **25**: 69–81.
59. Secombes CJ. Enhancement of fish phagocyte activity. *Fish Shellfish Immunol* 1994; **4**: 421–436.
60. Fujii T. Antibody-enhanced phagocytosis of lamprey polymorphonuclear leucocytes against sheep erythrocytes. *Cell Tissue Res* 1981; **219**: 41–51.
61. Griffin BR. Opsonic effect of rainbow trout (*Salmo gairdneri*) antibody on phagocytosis of *Yersinia ruckeri* by leukocytes. *Dev Comp Immunol* 1983; **7**: 253–259.
62. Sakai DK. Opsonization of fish antibody and complement in the immune phagocytosis by peritoneal exudate cells isolated from salmonid fishes. *J Fish Disease* 1984; **7**: 29–38.
63. Michel C, Gonzales R, Avraemeas S. Opsonizing properties of natural antibodies of rainbow trout, *Oncorhynchus mykiss* (Walbaum). *J Fish Biol* 1990; **37**: 617–622.
64. Chung S, Secombes CJ. Analysis of events occurring within teleost macrophages during the respiratory burst. *Comp Biochem Physiol* 1988; **89B**: 539–544.
65. Schoor WP, Plumb JA. Induction of nitric oxide synthase in channel catfish *Ictalurus punctatus* by *Edwardsiella ictaluri*. *Dis Aquat Org* 1994; **19**: 153–155.
66. Wang R, Neumann NF, Shen Q, Belosevic M. Establishment and characterisation of a macrophage cell-line from the goldfish. *Fish Shellfish Immunol* 1995; **5**: 329–346.
67. Hine PJ. The granulocytes of fish. *Fish Shellfish Immunol* 1992; **2**: 79–98.
68. Clem LW, Sizemore RC, Ellsaesser CF, Miller NW. Monocytes as accessory cells in fish immune responses. *Dev Comp Immunol* 1985; **9**: 803–809.
69. Verburg-van Kemenade BM, Weyto FAA, Flike G. Carp macrophages and neutrophils granulocytes secrete an IL-1-like factor. *Dev Comp Immunol* 1995: **19**: 59–70.
70. Elsaesser CF, Clem LW. Functionally distinct high and low molecular weight species of channel catfish and mouse IL-1. *Cytokine* 1994; **5**: 10–20.
71. Secombes CJ. The phylogeny of cytokines. In: Thomson A, ed. *The Cytokine Handbook* 2nd edn. London: Academic Press, 1994; 567–594.
72. Graham S, Secombes CJ. Cellular requirements for lymphokine secretion by rainbow trout *Salmo gairdneri* leucocytes. *Dev Comp Immunol* 1990; **14**: 59–68.
73. Jang SI, Hardie LJ, Secombes CJ. Elevation of rainbow trout *Oncorhynchus mykiss* macrophage respiratory burst activity in macrophage-derived supernatants. *J Leukocyte Biol* 1995; **57**: 943–947.
74. Dowding AJ, Maggs A, Scholes J. Diversity amongst the microglia in growing and regenerating fish CNS—immunohistochemical characterization using FL-1, an antimacrophage monoclonal antibody. *Glia* 1991; **4**: 345–364.
75. Horton JD, Ratcliffe NA. Evolution of immunity. In: Roitt I, Brostoff J, Male D, eds. *Immunology* 4th edn. London: Mosby, 1995; 15.1–15.22.
76. MacArthur JI, Thomson AW, Fletcher TC. Agents of leucocyte migration in the plaice, *Pleuronectes platessa* L. *J Fish Biol* 1985; **27**: 667–676.
77. Saggers BA, Gould ML. The attachment of micro-organisms to macrophages isolated from the tilapia *Oreochromis spilurus* Gunther. *J Fish Biol* 1989; **35**: 287–294.
78. Matsuyama H, Yano T, Yamakawa T, Nakao M. Opsonic effect of the third complement (C3) of carp (*Cyprinus carpio*) on phagocytosis by neutrophils. *Fish Shellfish Immunol* 1992; **2**: 69–78.
79. Pettitt TR, Rowley AF, Secombes CJ. Lipoxins are major lipoxygenase products of rainbow trout macrophages. *FEBS Lett* 1989; **259**: 168–170.
80. Pettitt TR, Rowley AF, Barrow SE, Mallet AI, Secombes CJ. Synthesis of lipoxins and other lipoxygenase products by macrophages from the rainbow trout, *Oncorhynchus mykiss*. *J Biol Chem* 1991; **266**: 8720–8726.
81. Rowley AF. Lipoxin formation in fish leucocytes. *Biochim Biophys Acta* 1991; **1084**: 303–306.
82. Rowley AF, Lloyd-Evans PL, Barrow SE, Serhan CN. Lipoxin biosynthesis by trout macrophages involves the formation of epoxide intermediates. *Biochemistry* 1994; **33**: 856–863.
83. Rowley AF, Knight J, Lloyd-Evans PL, Holland JW, Vickers PJ Eicosanoids and their role in immune modulation in fish—a brief overview. *Fish Shellfish Immunol* 1995; **5**: 549–567.
84. Ford-Hutchinson AW, Gresser M, Young RN. 5-Lipoxygenase. *Annu Rev Biochem* 1994; **63**: 383–417.
85. Knight J. Distribution and function of lipoxins and other eicosanoids in the rainbow trout, *Oncorhynchus mykiss*. PhD Dissertation, University of Wales, 1994.
86. Hendrick RP, MacConnell E, De Kinkelin P. Proliferative kidney disease of salmonid fish. *Annu Rev Fish Disease* 1993; **3**: 277–290.
87. Rainger GE, Rowley AF, Pettitt TR. Effect of inhibitors of eicosanoid biosynthesis on the immune reactivity of the rainbow trout, *Oncorhynchus mykiss*. *Fish Shellfish Immunol* 1992; **2**: 143–154.
88. Hunt TC, Rowley AF. Leukotriene $B_4$ induces enhanced migration of fish leucocytes *in vitro*. *Immunology* 1986; **59**: 563–568.
89. Sharp GJE, Pettitt TR, Rowley AF, Secombes CJ. Lipoxin-induced migration of fish leukocytes. *J Leukocyte Biol* 1992; **51**: 140–145.
90. Lee TH, Horton CE, Kyan-Aung U, Haskard D, Crea AEG, Spur BW. Lipoxin $A_4$ and lipoxin $B_4$ inhibit chemotactic responses of human neutrophils stimulated by leukotriene $B_4$ and *N*-formyl-L-methionyl-L-leucyl-L-phenylalanine. *Clin Sci* 1989; **77**: 194–203.
91. Soyombo O, Spur BW, Lee TH. Effects of lipoxin $A_4$ on chemotaxis and degranulation of human eosinophils stimulated by platelet-activating factor and *N*-formyl-L-methionyl-L-leucyl-L-phenylalanine. *Allergy* 1994; **49**: 230–234.
92. Colgan SP, Serhan CN, Parkos CA, Delp-Archer C, Madara JL. Lipoxin $A_4$ modulates transmigration of human neutrophils across intestinal epithelial monolayers. *J Clin Invest* 1993; **92**: 75–82.
93. Papayianni A, Serhan CN, Phillips ML, Rennke HG, Brady HR. Transcellular biosynthesis of lipoxin $A_4$ during adhesion of platelets and neutrophils in experimental immune complex glomerulonephritis. *Kidney Int* 1995; **47**: 1295–1302.
94. Knight J, Lloyd-Evans PL, Rowley AF, Barrow SE. Effect of lipoxins and other eicosanoids on phagocytosis and intracellular calcium mobilisation in rainbow trout (*Oncorhynchus mykiss*) leukocytes. *J Leukocyte Biol* 1993; **54**: 518–522.
95. Secombes CJ, Ashton I. Personal communication.

ACKNOWLEDGEMENTS. I am grateful to Professor N.A. Ratcliffe and Drs C.J. Secombes, R. Pipe, M. Page, E.A. Dyrynda, J. Knight and P.J. Vickers for allowing me to refer to their unpublished work and for their advice and helpful discussions. The results presented in this review from the author's laboratory were sup-ported by the Science & Engineering Research Council (GR/G/05179) and the Agricultural and Food Research Council (AG58/510).

**Received 1 November 1995; accepted 10 November 1995**
